# The global response to HIV/AIDS in sub-Saharan Africa: achievements, challenges, and perspectives for the future

**DOI:** 10.3389/fpubh.2025.1665666

**Published:** 2025-10-31

**Authors:** Stefano Orlando, Loredana Andreea Silaghi, Mariagrazia Cicala, Mathambo William Lowole, Cacilda Massango, Roberto Lunghi, Hawa Sangarè Mamary, Fausto Ciccacci, Paola Scarcella

**Affiliations:** ^1^Department of Biomedicine and Prevention, Università degli Studi di Roma Tor Vergata, Rome, Italy; ^2^Community of Sant'Egidio, DREAM Programme, Lilongwe, Malawi; ^3^Community of Sant'Egidio, DREAM Programme, Maputo, Mozambique; ^4^Community of Sant'Egidio, DREAM Programme, Rome, Italy; ^5^Libera Università Maria Ss. Assunta (LUMSA), Rome, Italy

**Keywords:** HIV, health financing, sub-Saharan Africa, differentiated service delivery (DSD), HIV inequalities

## Abstract

Over four decades, the global response to HIV/AIDS has transformed the disease into a manageable chronic condition, driven by advances in antiretroviral therapy, global financing, and ambitious targets like the 90-90-90 and 95-95-95 goals. Yet HIV remains a major challenge, especially in sub-Saharan Africa, which bears the highest burden worldwide. While AIDS-related mortality has declined, the number of people living with HIV continues to grow, placing significant strain on health systems and financial resources. Persistent challenges include high rates of new infections, late diagnoses, inequalities in access to care, and barriers faced by key populations. Emerging issues such as drug resistance and declining political commitment risk reversing progress. Investments in HIV have yielded broader benefits for health systems, supporting integration with services for tuberculosis and noncommunicable diseases. This review traces the global HIV response, analyses current epidemiological and economic trends, and highlights strategic priorities to sustain progress. Key strategic directions include reducing costs and expanding access to advanced diagnostics and antiretroviral therapy; prioritizing comprehensive, high-quality care over simplified delivery models that risk compromising diagnostic accuracy and fostering drug resistance; integrating HIV programs with services for other diseases, including noncommunicable diseases; strengthening surveillance and management of HIV drug resistance; and sustaining the visibility of HIV while addressing the needs of marginalized populations.

## Introduction

The first case of AIDS was diagnosed in 1981, and the Human Immunodeficiency Virus (HIV) was first identified in 1983 (1). Since then, approximately 88.4 million people have been infected with the virus, and an estimated 42.3 million have died as a result of HIV-related illnesses (2). In 1996, the United Nations established the Joint United Nations Programme on HIV/AIDS (UNAIDS) to coordinate the global response to the pandemic (3). The fight against HIV/AIDS rapidly became a key international priority, initially reflected in the Millennium Development Goals (MDGs)—specifically Goal 6, which aimed to “combat HIV/AIDS, malaria, and other diseases”—and later reaffirmed in the Sustainable Development Goals (SDGs) under Target 3.3, which commits to “end the epidemics of AIDS, tuberculosis (TB), malaria and neglected tropical diseases and combat hepatitis, water-borne diseases and other communicable diseases.”

Despite decades of progress, the burden of HIV remains substantial. In 2023, just years away from the 2030 SDG deadline, there were 1.3 million new infections, and 39.9 million people were living with HIV worldwide (4). Paradoxically, at this critical juncture, signs of waning political commitment have emerged: some donor countries have announced plans to scale back their support, signaling an apparent shift toward an exit strategy rather than renewed momentum to end AIDS with an expected dramatic impact on HIV response (5, 6).

The aim of this review is to examine the global response to AIDS through a historical lens, assessing the progress achieved over the past four decades, outlining the major challenges that persist today, and identifying strategic directions for the future. By tracing the evolution of global efforts—particularly in sub-Saharan Africa, the region most affected by the epidemic—this paper seeks to inform ongoing debates on sustainability, equity, and innovation in the fight to end AIDS.

### Country selection and scope

For the in-depth narrative, we selected case-study countries *a priori* based on: (i) epidemic burden (high adult prevalence and/or large numbers of people living with HIV), (ii) availability of recent, disaggregated data on the testing–treatment–viral suppression cascade and on financing, and (iii) documentation of service-delivery models relevant to policy transfer. This approach yields a set weighted toward eastern and southern Africa (high prevalence) while including at least one western/central African (WCA) example (Guinea) that illustrates a lower-prevalence, resource-constrained context.

## Historical context and global response to HIV/AIDS

### Milestones in the global response

The global HIV epidemic reached its peak incidence in 1995, with an estimated 3.3 million new infections occurring worldwide (4). In response, 1996 marked a pivotal year in the global fight against HIV/AIDS, characterized by significant institutional and therapeutic advancements. On the institutional front, the United Nations Economic and Social Council (ECOSOC) established the UNAIDS, aiming to coordinate international efforts and to unify strategies for ending the epidemic (7). Concurrently, a major therapeutic milestone was achieved with the introduction of combined Antiretroviral Therapy (ART) (7). This approach shifted treatment paradigms by utilizing multiple antiretroviral agents to inhibit HIV replication at different stages of the viral life cycle. Compared to previous monotherapies, combined ART significantly improved viral suppression, reduced disease progression, and transformed HIV infection into a manageable chronic condition (8).

Building on these efforts, the early 2000s saw the establishment of global financing initiatives aimed at expanding access to HIV prevention and treatment. In 2002, The Global Fund to fight AIDS, TB, and malaria was created to mobilize and disburse resources to countries most affected by these diseases (9). In 2003, the United States launched the President’s Emergency Plan for AIDS Relief (PEPFAR), which has since become one of the largest international health initiatives dedicated to a single disease (7).

These actions contributed to widespread success in terms of HIV treatment worldwide. According to estimates from the World Health Organization’s Global Health Observatory, global ART coverage among people living with HIV (PLHIV) has increased substantially over the past decade, rising from 24% (19–28%) in 2010 to 77% (61–89%) in 2023 (10). Significant progress has been observed particularly in sub-Saharan Africa, where the widespread scale-up of ART services has contributed to a marked decline in AIDS-related mortality both globally and regionally, shifting life expectancy at birth from 56.3 years in 2010 to 61.1 years in 2023 (11).

Such prompt response paved the way for more ambitious goals. In 2014, UNAIDS established the 90-90-90 targets as part of its strategy to end the AIDS epidemic as a public health threat. These targets aimed for 90% of all PLHIV to know their HIV status; 90% of those diagnosed to receive sustained ART; and 90% of those on treatment to achieve viral suppression. In subsequent years, these goals were elevated to higher levels, known as the 95-95-95 targets, reflecting advances in testing, treatment, and care. While these targets have not yet been fully reached at the global level, significant progress has been achieved. Globally, as of 2023, approximately 86% of PLHIV knew their status, 89% of those diagnosed were on ART, and 93% of those on treatment had achieved viral suppression (11). Considering these achievements, AIDS represents the first disease in history that prompted a coordinated global response that has proven highly effective.

Indeed, modeling analyses conducted by the Global Fund compare the substantial progress in reducing AIDS-related mortality and new infections over the past 25 years, and the possible trajectory of the epidemic without such a unified international effort. According to the analyses, AIDS-related deaths in countries where the Global Fund invests were reduced by 73% since 2002, and HIV infections by 61% since 2003 (9). While it remains challenging to determine the precise number of lives saved worldwide, the Global Fund estimates that interventions financed through its programs have saved approximately 65 million lives since 2002 (9). Including the impact of PEPFAR, national efforts, and other global initiatives, we could estimate that up to 100 million lives have been saved globally.

### Economic resources and health system strengthening

The scale of the global and coordinated response to AIDS can also be measured in terms of financial resources mobilized and invested over the past decades. Since 2010, funding for the AIDS response in low- and middle-income countries (LMICs) has consistently reached approximately 20 billion USD annually (9). Roughly half of these resources have been directed toward Eastern and Southern Africa, reflecting both the high burden of HIV in the region and the prioritization of interventions where the epidemic’s impact has been most severe (11).

It is worth noticing that the impact of financial investments in HIV programs varies widely across countries, due to local economic conditions and health system infrastructure. Table 1 reports, for each country, overall economic size (GDP) and the level of HIV programme expenditure (total, domestic + external), alongside three alignment ratios that aid comparison: HIV expenditure as a share of total health expenditure, as a share of government health expenditure, and as a share of GDP. Columns 4–5 show how much of national income is devoted to health overall and to public health, while columns 6–8 indicate the priority and fiscal exposure of the HIV response within the health sector and the wider economy. Percentages are computed against the corresponding aggregates for the same reference year as the HIV expenditure. Malawi serves as a remarkable case study. The country’s total spending on HIV/AIDS interventions in 2022 amounted to approximately 127% of the country’s entire public health expenditure and nearly 2% of its national GDP. This overview highlights the substantial scale of investment relative to national resources.

**Table 1:** Health resources and HIV financing across countries—standardized indicators for cross-country comparison.

Country	HIV expenditure (US$, total)	GDP (US$ millions)	Total health expenditure (% of GDP)	Government health expenditure (% of GDP)	HIV expenditure (% of total health expenditure)	HIV expenditure (% of government health expenditure)	HIV expenditure (% of GDP)
Guinea	27.264.405	22.199.41	3.80%	0.70%	3.23%	17.55%	0.12%
Kenya	764.057.566	108.038.59	4.50%	2.20%	15.72%	32.15%	0.71%
Malawi	227.510.308	12.712.15	7.40%	1.40%	24.19%	127.84%	1.79%
Mozambique	557.780.694	20.954.22	9.10%	2.60%	29.25%	102.38%	2.66%
South Africa	2.329.049.277	380.699.27	8.30%	5.00%	7.37%	12.24%	0.61%
Uganda	538.920.125	48.768.96	4.70%	1.10%	23.51%	100.46%	1.11%
Zambia	439.789.941	27.577.96	6.60%	2.80%	24.16%	56.95%	1.59%
Zimbabwe	129.516.626	35.231.37	2.80%	0.90%	13.13%	40.85%	0.37%

Data sources: UNAIDS (51); World Bank Group (53).

Finally, it is worth considering how the benefits of these investments extend well beyond HIV/AIDS-specific interventions. Indeed, they contribute to broader health system strengthening in terms of capacity building, workforce retention, and infrastructure. Indeed, funds have supported the recruitment, training, and retention of health professionals, helping to mitigate brain drain and enhancing capacity for both HIV services and general healthcare delivery. Similarly, infrastructure investments have established numerous molecular laboratories, significantly improving diagnostic and treatment capabilities across a range of health conditions.

## Fundamental issues and current challenges

Despite the significant achievements of the past decades, several critical issues remain unresolved and pose substantial challenges for the future. These must not be underestimated, particularly in sub-Saharan Africa, where the epidemic continues to exert a heavy toll.

While the adult HIV prevalence rate has plateaued globally at approximately 0.7%, this figure remains higher than in 1996—the year ART was introduced—when prevalence stood at 0.6%. Moreover, the absolute number of PLHIV continues to rise. As of 2023, an estimated 39.9 million people were living with HIV globally—an increase of 400,000 compared to the previous year 21/08/2025 23:52:00. In several countries—Kenya, Mozambique, South Africa, Uganda, the United Republic of Tanzania, Zambia, and Zimbabwe—the number of PLWH exceeds one million, underscoring the continued severity of the epidemic in the region (12).

The growth in absolute numbers is partly attributable to a positive trend: the substantial decline in AIDS-related mortality. However, this demographic success has created a new challenge: the increasing number of individuals who require long-term care and sustained access to ART. This growing cohort places a significant burden on already stretched health systems and financing mechanisms. For example, in Mozambique, where approximately 2.4 million people are living with HIV, assuming an estimated annual cost per patient for first-line ART, clinical monitoring, and health personnel of USD 250 as calculated for countries in the area (13–15). This excludes the additional costs associated with second-line therapies and the management of HIV-related comorbidities. Based on these estimates, the annual financial requirement to sustain HIV care exceeds USD 600 million, or roughly 3% of the country’s GDP. For comparison, Mozambique’s entire public health expenditure currently accounts for only 2.6% of GDP. In other words, HIV/AIDS alone would require more financial resources than the entire health sector currently receives, raising urgent questions about the sustainability of current funding models and the need for innovative approaches to financing HIV responses.

A critical area that continues to demand urgent attention is the prevention of new HIV infections. Among the most notable successes achieved thus far is the prevention of mother-to-child transmission (PMTCT). In many parts of the world, the number of children born with HIV to mothers living with the virus has dropped to zero—marking significant progress toward the global target known as “Getting to Zero” (i.e., zero new infections). However, this goal remains far from being realized in several high-burden countries. In Guinea, for example, the vertical transmission rate remains as high as 20%, while in Mozambique, it hovers around 10%. As a result, the number of children aged 0–14 living with HIV in Mozambique alone is still estimated at 150,000, highlighting critical gaps in the implementation and scale-up of PMTCT programs in certain contexts (16).

Another major innovation in HIV prevention has been the introduction of pre-exposure prophylaxis (PrEP), and more recently, the development of long-acting formulations, such as biannual injectable agents like lenacapavir (17). These advances represent a promising step toward reducing HIV incidence, particularly among populations at substantial risk. However, significant barriers to access and sustained adherence to PrEP remain—especially among the most vulnerable and marginalized populations, including adolescent girls and young women (AGYW), men who have sex with men, sex workers, and people who inject drugs (18, 19).

A further critical insight that emerges from a careful reading of the epidemiological data is that reducing the total number of PLHIV is not possible without a substantial decline in new infections. Over the past decade, one finding has become increasingly clear: the most effective form of prevention is viral suppression through ART. This principle is captured by the now widely recognized message “U = U” (Undetectable = Untransmittable), which affirms that individuals who maintain an undetectable viral load through consistent ART do not transmit the virus (20–22). In essence, the most effective strategy to curb new infections is to ensure universal access to HIV testing and the immediate initiation of treatment for all those who test positive. This is the foundation of the UNAIDS 95-95-95 targets, which aim for 95% of PLHIV to be diagnosed, 95% of those diagnosed to be on ART, and 95% of those on treatment to achieve viral suppression. However, as a cascade model, these targets imply that with each step, approximately 5% of individuals may be lost to follow-up or remain unserved. Consequently, even if the 95-95-95 goals are met, an estimated 14% of all PLHIV would remain potentially infectious (23). In a country like Kenya, where approximately 1.4 million people live with HIV, this translates to nearly 196,000 individuals, clearly showing the magnitude of this barrier to achieving HIV elimination.

Moreover, delayed treatment initiation—even among individuals who eventually achieve viral suppression—extends the period during which they remain infectious. This underscores the importance not only of achieving the targets, but of doing so rapidly. Unfortunately, late diagnosis remains a significant challenge, with many individuals only receiving a diagnosis once AIDS has already progressed to an advanced stage, often defined by CD4 counts below 200 cells/mm^3^ (24, 25). Despite sustained case-finding gains, late presentation persists. Using the WHO definition of advanced HIV disease (AHD) (CD4 < 200 cells/mm^3^ or WHO stage 3/4), recent analyses from sub-Saharan Africa indicate that ~15–30% (or higher) of adults initiating or re-initiating ART present with AHD, with marked heterogeneity by country and population group. A 2024–2025 synthesis estimates ~1.8–1.9 million people in the region are living with AHD at any given time, with men and older adults over-represented (26, 27). Although comprehensive data are lacking in many countries, one recent study conducted in Kenya and Malawi reported that 9.7% of people newly diagnosed with HIV had late-stage disease. The figure rose to 15.3% among men, suggesting important gender disparities in healthcare access and testing behavior (28).

### Inequality and epidemiological disparities

**Figure 1:**
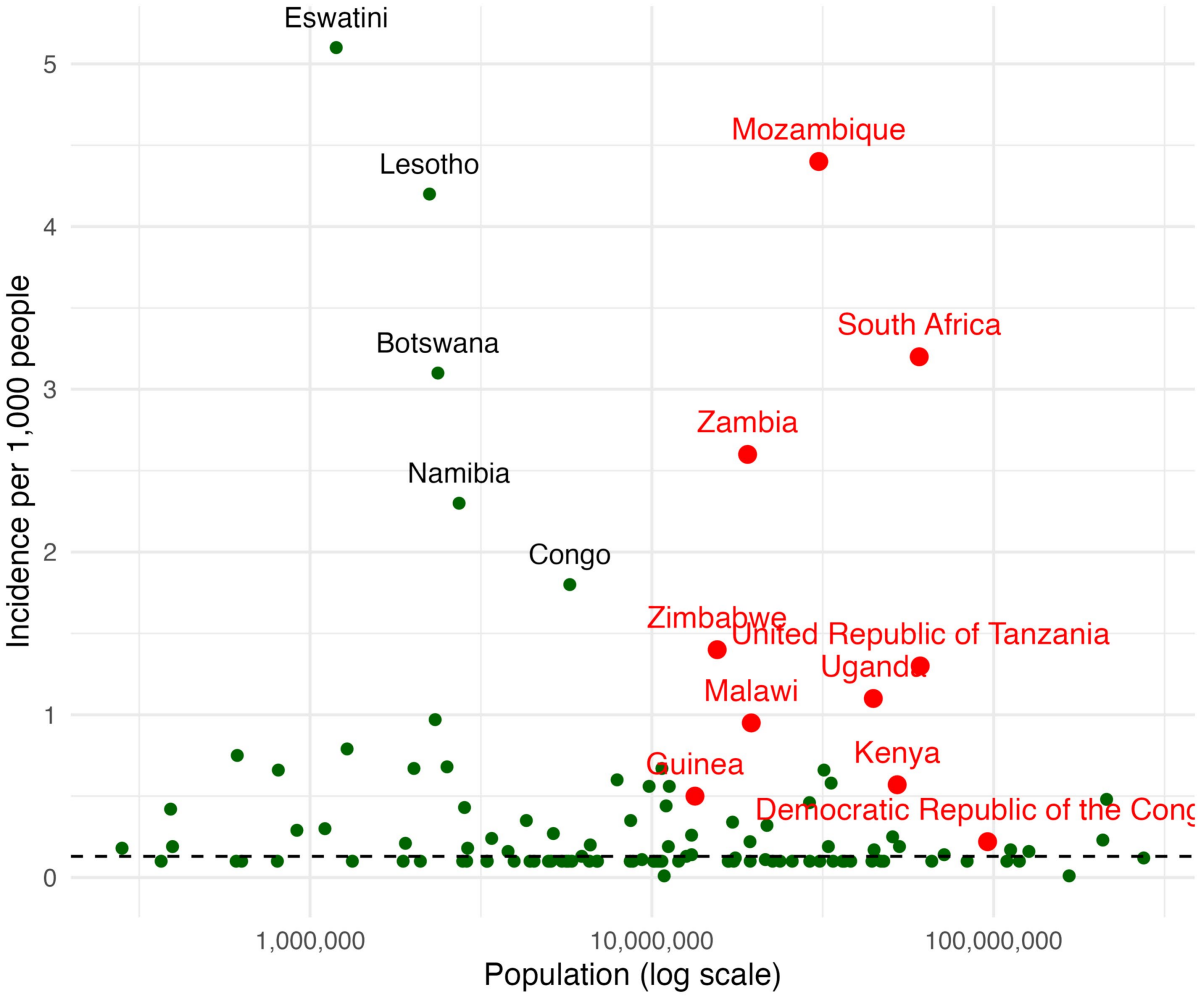
HIV incidence and national population size, global by country.

**Figure 2:**
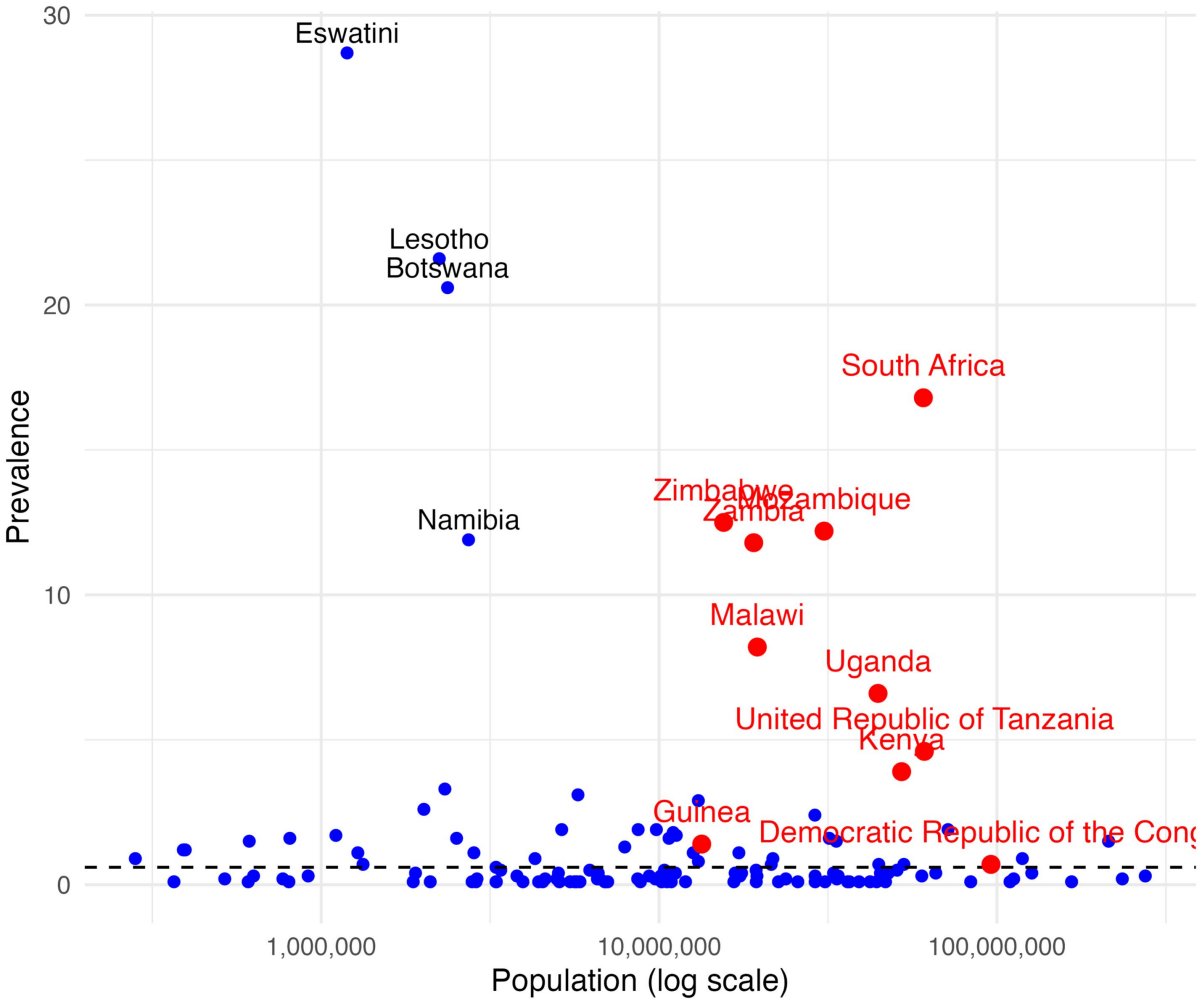
HIV prevalence and national population size, global by country.

Another fundamental issue is the persistence of inequalities, which significantly undermine the global response to HIV (29). A critical point to bear in mind is that a declining global average in new infections can conceal wide disparities across countries and populations. As illustrated in Figures 1, 2, which plot HIV prevalence and incidence against national population size (on a logarithmic scale), the data show substantial variation around the global mean. Several large sub-Saharan African countries remain far behind in reaching the targets for HIV prevention and control. For example, Mozambique, South Africa, Zambia, Zimbabwe, Tanzania, Malawi and Uganda stand out for being big countries in terms of population size, with an extremely high prevalence or incidence rates, well above global averages.

Alongside disparities between nations, substantial inequalities exist within countries themselves. One critical axis of disparity is the difference between urban and rural areas, where access to HIV testing, treatment, and care often varies markedly (30). Urban centres typically benefit from better infrastructure, higher availability of health services, and greater integration into health surveillance systems. By contrast, rural populations frequently face barriers such as long travel-time to HIV services, geographic isolation, shortages of trained health personnel, and weaker health system capacity, all of which can limit access to timely diagnosis and treatment (11, 31). In sub-Saharan Africa, these disparities translate into negative outcomes in all stages of HIV care (32). A further concern is that sentinel surveillance systems concentrate on urban health facilities and selected sites. Such data often fail to capture the full epidemiological profile of rural regions, leading to potential underestimation of HIV prevalence and incidence in underserved areas (33, 34). This gap has critical implications for program planning and resource allocation, risking inequities in the distribution of prevention and treatment services.

Moreover, key populations—including AGYW, sexual minority men, sex workers, people who inject drugs, transgender and gender diverse individuals, prisoners, and their partners—continue to experience disproportionate vulnerability to HIV infection. These groups often face overlapping challenges related to stigma, discrimination, criminalization, and social marginalization, all of which exacerbate the barriers to accessing prevention, testing, treatment services, and adherence to care (29). In sub-Saharan Africa, HIV infection incidence among AGYW increased to four out of five new infections, compared to the three out of five in 2017 (11). Similarly, children below 14 have a 35% lower HIV testing, treatment initiation, and suppression rate compared to the older ones (29). Systemic inequalities worldwide, such as socioeconomic disparities, colonial history, racism, gender inequality, violence, and punitive laws further exacerbate barriers to care (29).

Together, these inter-country and intra-country disparities underscore that while global progress against HIV has been substantial, it remains uneven and fragile. A nuanced understanding of both geographical and social inequalities is therefore essential for designing effective strategies that ensure no populations are left behind in the global effort to end the HIV epidemic.

## Strategic directions and potential developments

### Cost reduction and enhanced accessibility

Looking ahead, emerging strategic directions and potential developments could significantly influence the future of the HIV response.

First, the reduction of ART and diagnostic costs and the enhanced access to testing and care. Throughout recent years, there has been a sustained effort to reduce the cost of ART and facilitate its widespread adoption, particularly in LMICs (7). In the early 2000s, the annual cost of first-line ART was prohibitively high for most health systems in resource-limited settings. In 2000, the median price for a first-line combination ART regimen in LMICs was approximately 10,000 USD per patient per year (35). In 2014, the cost for a generic first-line treatment dropped to about 100 USD per adult patient per year, driven largely by the introduction of generic antiretroviral drugs, global policy advocacy, and international financing mechanisms such as the Global Fund and PEPFAR (35). These reductions transformed ART from an inaccessible intervention into a cornerstone of HIV treatment worldwide. Parallel reductions have occurred in the costs of essential laboratory tests, such as viral load monitoring, which are crucial for optimizing patient management and assessing treatment efficacy (36).

The same path may be undertaken by addressing the availability and affordability of advanced diagnostic tools, such as HIV drug resistance testing. While the latter is part of the standard of care in high-income countries, it remains largely unavailable in many low-income settings due to high costs, lack of laboratory infrastructure, and limited technical expertise (37). Addressing these disparities is essential, as the lack of routine resistance testing in resource-limited settings can result in delayed detection of treatment failure, the spread of resistant HIV strains, and increased costs associated with switching to more expensive second- or third-line regimens (38).

### Balancing service simplification and quality of care

Second, the long-term sustainability of the quality of care needs to be preserved. The implementation of differentiated service delivery (DSD) has simplified HIV management and improved access to testing and care, tailoring services according to the patients’ needs to ultimately ensure more effective and accessible treatment (39). While it promoted a widespread access to treatment, there remains a critical need to ensure that simplification does not come at the expense of quality. The use of simplified protocols may in fact reduce the frequency or scope of laboratory monitoring, clinical assessments, or access to integrated prevention and treatment services, potentially resulting in delayed detection of treatment failure (40). Another particular risk is the emergence and spread of HIV drug resistance. Indeed, limited virological monitoring and the absence of routine drug resistance testing in many LMICs increase the likelihood that treatment failure goes unrecognized, enabling the accumulation of resistant viral strains. This challenge not only threatens individual patient outcomes but also poses broader public health risks, potentially undermining the efficacy of first-line regimens and increasing the need for more expensive treatments (38). To sustain long-term progress against HIV, it is therefore essential to strike a balance between simplification and preservation of comprehensive care models that ensure quality, equity, and sustainability. Such comprehensive care encompasses the integration of ART provision, routine viral load monitoring, resistance testing where feasible, psychosocial support, and integration of services for co-infections and comorbidities.

### Attention to marginalized issues and populations

While absolute numbers are critical in understanding public health challenges from a wider perspective, it is important not to overlook the issue of marginality. Barriers to treatment may disproportionately affect minority or marginalized populations, resulting in challenges that involve hundreds of thousands of individuals worldwide. Neglecting these populations has profound ethical and epidemiological implications. On the ethical side, ensuring equitable access to effective treatment and care is fundamental to the principles of justice and human rights enshrined in global health frameworks. Epidemiologically, failing to address the needs of smaller, high-risk populations can undermine broader public health objectives. For instance, undiagnosed or untreated individuals with drug-resistant HIV strains not only suffer worse health outcomes but also pose a risk of transmitting resistant virus within communities, potentially reversing progress in HIV control (11). Moreover, persistent gaps in treatment access among key and marginalized populations can hinder efforts to achieve the 95-95-95 targets and ultimately threaten the goal of ending AIDS as a public health threat. Addressing the needs of these groups is therefore essential as a strategic component of effective epidemic control.

### Co-benefits of continued investment in HIV/AIDS and integration with other diseases programs, including NCDs

In both health and economics, the core challenge is not merely identifying needs or determining what ought to be done but understanding how competing needs vie for the same limited resources. In this sense, advancing and strengthening the HIV response is often perceived as requiring resources that must be diverted from other health priorities—or even from other sectors altogether. This has led to long-standing criticisms of disease-specific (vertical) programs, particularly those focused on HIV/AIDS, for allegedly creating siloed approaches that drain funding and attention from broader health system strengthening and the prevention and treatment of other conditions (41–43).

At the international level, several major donors have recently expressed concern about the sustainability of maintaining the current scale of HIV investments, particularly given the growing number of global health priorities competing for attention and funding—ranging from pandemic preparedness to noncommunicable diseases (NCDs), mental health, and universal health coverage (44, 45). However, evidence accumulated over the past decades challenges the assumption that HIV investments necessarily come at the expense of other health priorities. Rather than a zero-sum game, the experience of the HIV response often demonstrates a multiplier effect. Investments targeted at one disease can yield system-wide benefits (46–48).

A key area where this dynamic is most visible is the integration of HIV services with those for other comorbid conditions, particularly TB and NCDs. These co-morbidities are increasingly prevalent even in low-income settings and place a growing burden on fragile health systems. Data from a recent study conducted in a semi-rural region of Kenya, revealed that approximately 40% of patients receiving HIV care also presented with at least one comorbidity. These findings underscore the critical need for early diagnosis, long-term treatment, and continuous monitoring—capabilities that have been developed and refined through decades of HIV program implementation (49).

The skills, infrastructure, and systems established for HIV care—such as community-based outreach, chronic disease management, laboratory networks, and supply chains—are highly relevant and adaptable to the management of other chronic conditions. Incorporating screening, diagnosis, and treatment of comorbidities into HIV programs could therefore generate substantial health benefits and potentially lead to significant downstream cost savings. In this light, HIV investments should not be viewed as a drain on the system, but rather as a strategic platform for broader health system integration and reform. Rather than a source of competition, they represent an opportunity for co-benefit and synergy.

## Discussion

Over four decades, the global response to HIV/AIDS—particularly in sub-Saharan Africa—has transformed a once invariably fatal infection into a chronic, manageable condition for millions (8). Through the rapid expansion of ART, the establishment of global financing instruments such as the Global Fund and PEPFAR, and the adoption of unified targets like 90-90-90 and 95-95-95, the HIV response has achieved an unprecedented scale and reach. Mortality has declined dramatically, incidence has fallen in many settings, and millions of lives have been saved. Moreover, the HIV response has yielded co-benefits far beyond its original mandate: strengthening health systems, expanding laboratory networks, and serving as a platform for integrated service delivery.

Yet the success of the HIV response should not obscure its fragility. Several enduring challenges threaten to undermine or even reverse progress. The growing number of PLHIV—now approaching 40 million—reflects both the success of ART and the limitations of current prevention strategies (44, 47). While viral suppression remains the most effective method of prevention, late diagnosis, dropouts across the care cascade, and barriers to treatment access—especially among marginalized populations—continue to sustain transmission. Diagnostic delays, particularly in men and rural populations, compromise both individual outcomes and public health goals. Meanwhile, persistent inequalities—between and within countries—reveal that aggregate gains often mask uneven progress. These disparities are amplified by structural drivers of vulnerability: gender inequality, poverty, stigma, criminalization, and geographic inaccessibility (50).

In settings where distance to laboratories drives delayed clinical action, a pragmatic package of near−/point-of-care tools can materially narrow disparities. Priority components include near-POC viral load and EID (to compress time-to-result and enable same-day or next-visit decisions), CD4-based triage for advanced HIV disease alongside cryptococcal antigen (CrAg) lateral-flow testing and urine LAM for TB in eligible patients, and HIV self-testing with assisted linkage for populations that under-utilize facility testing (particularly men in rural areas). Program enablers—hub-and-spoke sample referral for when true POC is not feasible, digital connectivity for rapid result transmission and cohort monitoring, external quality assessment, and secure supply of test kits and OI medicines—are essential to translate access into improved viral suppression and survival. These elements are feasible within primary-care platforms and align with our emphasis on quality-preserving simplification rather than cost-saving at the expense of outcomes.

**Figure 3:**
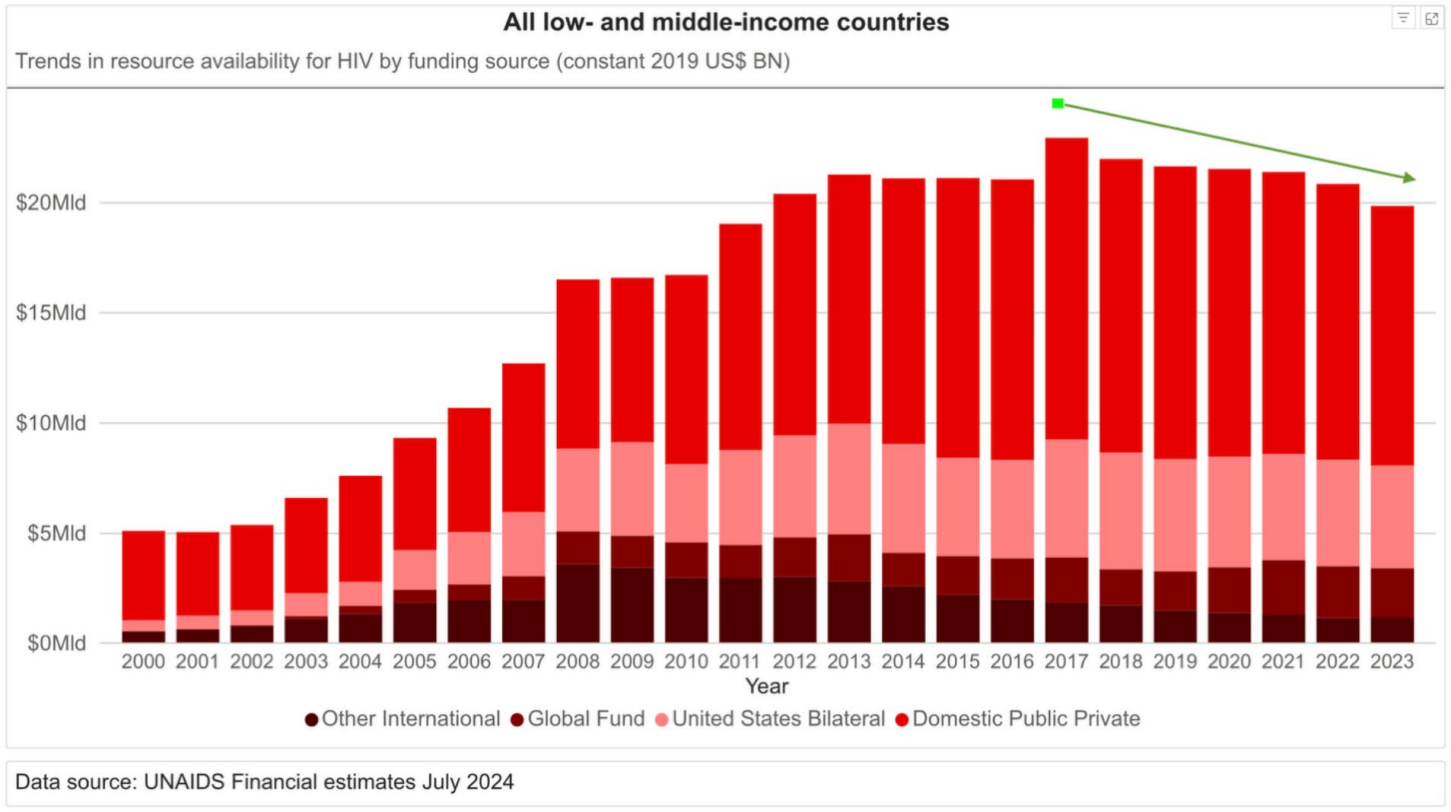
Annual trends in resource availability for HIV by funding source (constant 2019 USD BN) in all low- and middle-income countries.

Looking forward, several imperatives emerge. First, sustained and predictable financing is essential. Despite compelling evidence of impact and cost-effectiveness, international support for HIV/AIDS is plateauing or even declining, threatening the continuity of care for millions (47). Figure 3 presents annual trends in global resources allocated to the HIV response, based on data reported by UNAIDS website (51). The graph clearly illustrates a sustained increase in funding from 2000 to 2017, but beginning around 2017, a downward trend becomes evident, marking a shift in global financing priorities. This decline raises significant concerns about the sustainability of current HIV programs, particularly in countries that remain heavily dependent on external aid. The reversal of earlier funding gains threatens not only to slow progress but also to jeopardize the gains already achieved, including the scaling-up of antiretroviral therapy, testing, and prevention services.

In low-income countries such as Malawi or Mozambique, where HIV expenditures already exceed total public health spending, even minor funding reductions can precipitate service disruptions with cascading consequences. The notion that HIV financing is a zero-sum drain on other priorities is not supported by evidence; instead, investments in HIV have demonstrated multiplier effects across health systems. The integration of HIV services with TB and NCDs management exemplifies a strategic path toward broader health system resilience.

**Figure 4:**
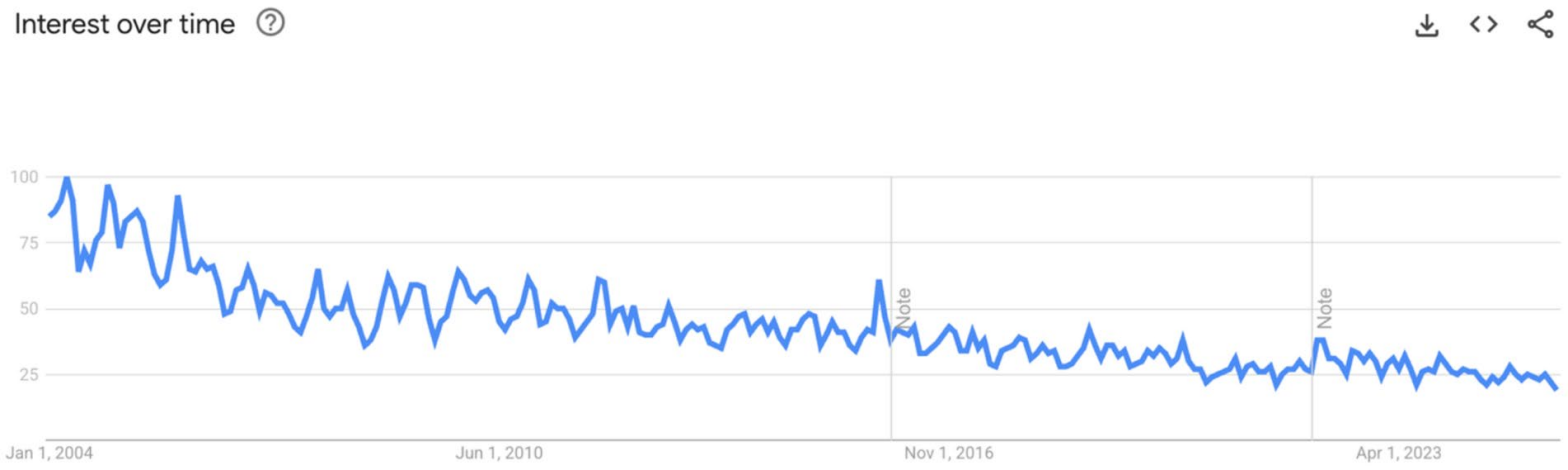
Google search trends for the terms *HIV* and *AIDS* from 2004 to 2024.

Second, the visibility of HIV/AIDS in global public discourse must be maintained. Trends in public attention, including online search interest, suggest a waning salience of HIV in the global health narrative, despite its continued burden and complexity. Figure 4 displays the Google search trends for the terms *“HIV”* and *“AIDS”* from 2004 to the present, illustrating a marked and sustained decline in public interest over time. While search volume is an imperfect proxy for societal engagement, it offers a useful indicator of how prominently a topic figures in public discourse (52). This decline in visibility may foster policy complacency and donor fatigue. Strategic communication and advocacy are therefore crucial to sustain momentum and resource mobilization, particularly in the face of competing global health priorities.

Finally, there is a moral dimension that cannot be overlooked. The global HIV response was born not only of scientific innovation and political will, but of solidarity and a recognition of shared humanity. The recent withdrawal and pausing of international HIV financing—notably the U.S. Government’s 2025 pause in foreign assistance disbursements affecting USAID/PEPFAR-implemented programmes, alongside cuts announced by other major donors—poses a material risk to epidemic control in high-burden countries. Modeling in The Lancet HIV (2025) suggests that, if unmitigated, 2025–2026 funding reductions could result globally in 4.4–10.8 million additional HIV infections and 0.77–2.9 million additional HIV-related deaths by 2030, with the largest absolute impact in eastern and southern Africa. Programmatically, the most immediate vulnerabilities are ARV and diagnostic procurement, viral load/EID testing capacity, advanced HIV disease (AHD) commodities (e.g., CrAg and urine LAM), and retention-critical human resources. Retreating from the commitment to sustained financing—precisely when the tools to end AIDS exist—would therefore be both ethically indefensible and pragmatically self-defeating (6). Leaving behind the most vulnerable populations, whether due to stigma, geography, or lack of profitability, undermines the very foundations of the public health enterprise. A failure to act decisively and inclusively risks reversing decades of progress and betraying the promise of universal health equity.

## Limitations

Our case-study set prioritizes data-rich, policy-illustrative contexts and is not exhaustive; to avoid unintended emphasis, we now explicitly flag WCA epidemiology and name large-burden WCA countries (e.g., Nigeria, Côte d’Ivoire) as comparators to the ESA cases.

In sum, the HIV response stands at a crossroads. Tremendous achievements have been made, but the path ahead is fraught with risk. The goal of ending AIDS as a public health threat by 2030 remains within reach—but only if the global community recommits to bold, equitable, and sustained action. Strategic investments, continued innovation, and unwavering attention to human rights must guide the final stretch of this historic journey.
